# Can IDO activity predict primary resistance to anti-PD-1 treatment in NSCLC?

**DOI:** 10.1186/s12967-018-1595-3

**Published:** 2018-08-06

**Authors:** Andrea Botticelli, Bruna Cerbelli, Luana Lionetto, Ilaria Zizzari, Massimiliano Salati, Annalinda Pisano, Mazzuca Federica, Maurizio Simmaco, Marianna Nuti, Paolo Marchetti

**Affiliations:** 1 Department of Clinical and Molecular Medicine, Sant’Andrea Hospital, Sapienza University of Rome, Via di Grottarossa 1035-1037, 00189 Rome, Italy; 2 Department of Radiological, Oncological and Pathological Sciences, Sapienza University of Rome, Rome, Italy; 3 Experimental Immunology Laboratory, Biochemistry Laboratory, IDI-IRCCS FLMM, Rome, Italy; 4 Department of Experimental Medicine, “Sapienza” University of Rome, Rome, Italy; 5 Department of Oncology, Università di Modena e Reggio Emilia, Policlinico di Modena, Modena, Italy; 6 Advanced Molecular Diagnostics Unit, Sant’Andrea Hospital, Sapienza University of Rome, Rome, Italy

**Keywords:** IDO, Immunotherapy, Nivolumab, Kynurenine, Anti-PD-1

## Abstract

**Background:**

Immune checkpoint inhibitors have revolutionized the treatment paradigm of highly lethal malignancies like advanced non-small cell lung cancer (NSCLC), demonstrating long-term tumour control and extended patient survival. Unfortunately, only 25–30% of patients experience a durable benefit, while the vast majority demonstrate primary or acquired resistance. Recently, indoleamine 2,3-dioxygenase (IDO) activity has been proposed as a possible mechanism of resistance to anti-PD-1 treatment leading to an immunosuppressive microenvironment.

**Methods:**

Pre-treatment serum concentrations of tryptophan (trp) and kynurenine (kyn) were measured by high-performance liquid chromatography tandem mass spectrometry in NSCLC patients treated with second-line nivolumab. The IDO activity was expressed with kyn/trp ratio. The associations between kyn/trp ratio and early progression, performance status (PS), age, sex, brain metastases, pleural effusion, progression free survival (PFS) and overall survival (OS) were analyzed using Spearman test and Mann–Whitney test.

**Results:**

Twenty-six NSCLC patients were included in our study; 14 of them (54%) presented early progression (< 3 months) to nivolumab treatment. The median value of kyn/trp ratio was 0.06 µg/ml and the median value of quinolinic acid was 68.45 ng/ml. A significant correlation between early progression and higher kyn/trp ratio and quinolinic acid concentration was observed (*p *= 0.017 and *p *= 0.005, respectively). Patients presenting lower values of kyn/trp ratio and quinolinic acid levels showed longer PFS (median PFS not reached versus 3 months; HR: 0.3; *p *= 0.018) and OS (median OS not reached vs 3 months; HR: 0.18; *p *= 0.0005).

**Conclusion:**

IDO activity, expressed as kyn/trp ratio, is associated with response to immunotherapy; in particular, higher kyn/trp ratio could predict resistance to anti-PD-1 treatment. These preliminary results suggest the possibility of using anti-PD-1 plus IDO inhibitor in those patients with high level of kyn/trp ratio.

## Background

In NSCLC, nivolumab showed a long-term benefit in a significant proportion of pretreated patients with 2-year overall survival (OS) of 23 and 29% in squamous and non-squamous histology, respectively, over-performing standard chemotherapy [1, 2]. However, more than two-thirds of NSCLC patients demonstrate primary or acquired resistance to PD-1 inhibition resulting in treatment failure [3]. Moreover, recent evidence suggested the existence of a novel pattern of disease progression referred to as hyper-progressive and characterized by a two-fold increase in tumour burden at first instrumental assessment following anti-PD-1 treatment and very poor OS [4]. Therefore, there is an urgent need for biomarkers able to predict treatment responsiveness and to identify those patients unlikely to respond to PD-1 blockade *in order to* candidate them to different treatment approaches. While some predictive factors of response to immune checkpoint inhibitors (ICI) have been proposed, among which PD-L1 expression/amplification, high tumor mutational burden, and mismatch repair gene defects, reliable biomarkers for treatment resistance are still lacking [3]. Indoleamine 2,3-dioxygenase (IDO) is a key enzyme catalyzing the first and rate-limiting step along the kynurenine (kyn) pathway of tryptophan (trp) metabolism outside the liver, which converts the essential amino acid l-trp to the main metabolite kyn [5]. Indoleamine 2,3-dioxygenase has been shown to act as immune checkpoint involved in peripheral immune tolerance since it is able to inhibit T-cells proliferation by starving them from trp to sensitize T-cells to apoptosis [6–11]. In NSCLC, augmented trp catabolism, resulting in higher kyn serum concentration, has been linked to more advanced stage at diagnosis, poorer prognosis and to a lesser likelihood of response to chemotherapy [12–14]. More interestingly, growing preclinical evidence suggests that increase in IDO activity can be involved in resistance to checkpoint inhibition [15] In the present study, we aimed to investigate whether IDO activity, expressed as kyn/trp ratio, can inform about response to nivolumab, particularly identifying those patients resistant to PD-1 blockade in a cohort of previously treated NSCLC.

## Methods

### Patients

Patients with stage IV NSCLC followed at our Institution from June 2016 to July 2017 were enrolled onto this retrospectively study. Inclusion criteria were: age > 18 years; histologically-documented diagnosis of NSCLN; Eastern Cooperative Oncology Group (ECOG) performance status ≤ 2; second-line treatment with anti-PD-1 nivolumab; adequate cardiac, pulmonary, renal, liver and bone marrow function; written informed consent. Exclusion criteria were: autoimmune disease; symptomatic interstitial lung disease and any other significant comorbidity; systemic immunosuppression; prior treatment with immune-stimulatory antitumor agents including checkpoint-targeted agents.

Nivolumab treatment was administered at a standard dose of 3 mg/kg every 2 weeks until disease progression or development of unacceptable toxicity. Radiological response was assessed with i-RECIST Criteria and classified according to disease control (complete response, partial response and stable disease) and progressive disease. All toxicity was graded according to the National Cancer Institute Common Terminology Criteria for Adverse Events (version 4.0) and toxicity assessments performed at day 1 of every cycle until the end of treatment. Progression-free survival (PFS) was defined as the time from patient registration on clinical trial until the first documented tumour progression or death from any cause. Overall survival (OS) was defined as the time from patient registration to death from any cause.

We defined as early progressors, patients experiencing disease progression within three months from the beginning of nivolumab. The study was conducted in accordance with good clinical practice guidelines and the declaration of Helsinki. The final version of the protocol was approved by the Institutional Ethics Committee.

### Kynurenine analysis

We evaluated serum levels of trp, kyn, and quinolinic acid by a modified liquid chromatography–tandem mass spectrometry (LC–MS/MS) method, [ADHD]. Serum samples were collected and stored at − 80 °C until analysis. Fifty μl of serum samples were deproteinized using 50 μl of Internal Standard (IS) solution (50 µM in TCA 4%), vortex-mixed and centrifuged at 14,000 rpm for 15 min. Twenty μl of clean upper layer were injected into chromatographic system. Chromatographic separation of analytes was performed using an Agilent Liquid Chromatography System series 1100 (Agilent Technologies, USA), on a biphenyl column (100 × 2.1 mm, Kinetex 2.6 μm Biphenyl, 100 Å, Phenomenex, CA, USA) equipped with a security guard precolumn (Phenomenex, Torrance, CA, USA). The mobile phase consisted of a solution of 0.1% aqueous formic acid (A) and 100% methanol (B); elution was performed at flow rate of 400 μl/min, using an elution gradient. The mass spectrometry method was performed on a 3200 triple quadrupole system Applied Biosystems, Foster City, CA, USA) equipped with a Turbo Ion Spray source. The detector was set in the positive ion mode. The instrument was set in the Multiple Reaction Monitoring (MRM) mode. Data were acquired and processed by the Analyst 1.5.1 Software.

### Statistical analysis

In the descriptive analysis, quantitative variables were described as mean and range, while qualitative variables as number and percentage.

PFS and OS were estimated using the Kaplan–Meier method, comparison between groups were made using the log-rank test, and Mantel-Cox method was used to generate hazard ratios (HRs) and 95% confidence intervals (CIs).

Correlation was estimated using Spearman’s rho and comparison was evaluated using the nonparametric Mann–Whitney U test.

To determinate factors associated with early progressors, multivariate logistic regression models were used. Results of multivariate analysis were expressed in odds ratio and 95% CIs. Statistical significance was set at p < 0.05. Statistical analyses were performed using IBM SPSS Statistics for Window Version 23.0 (Armonk, NY, USA) or GraphPad Prism (GraphPad, Inc, San Diego, CA, USA).

## Results

### Clinical characteristics

Twenty-six stage IV NSCLC patients treated with second-line nivolumab were enrolled in this study. Baseline clinicopathological characteristics of patients are summarized in Table 1. Among them, 19 patients had squamous-cell carcinoma and the remaining had non-squamous histology (6 adenocarcinoma and 1 undifferentiated tumour). Twenty patients were male (76.9%), and median age was 65 years. The median value of kyn/trp was 0.06 µg/ml and median value of quinolinic acid was 68.45 ng/ml.

**Table 1:** Main baseline characteristics of the study population

	N (%)	Serum kyn/trp ratio (µg/ml)	Serum quinolic acid concentration (ng/ml)
Age			
< 65	11 (42.3)	0.06 ± 0.03	74.47 ± 45.93
65+	15 (57.7)	0.08 ± 0.05	102.55 ± 72.99
Sex			
Male	20 (76.9)	0.08 ± 0.04	99.21 ± 69.37
Female	6 (23.1)	0.05 ± 0.02	62.22 ± 24.24
Histology			
Adenocarcinoma	6 (23.1)	0.09 ± 0.04	127.85 ± 59.78
Squamous-cell carcinoma	19 (73.1)	0.07 ± 0.04	79.69 ± 63.25
Undifferentiated tumor	1 (3.8)	0.04	76.2
ECOG PS			
0–1	25 (96.2)	0.07 ± 0.03	88.26 ± 63.54
> 1	1 (3.8)	0.18	151
Cerebral metastasis			
Yes	3 (11.5)	0.05 ± 0.04	91.54 ± 64.77
No	23 (88.5)	0.08 ± 0.04	84.03 ± 64.14
Pleural effusion			
Yes	6 (23.1)	0.07 ± 0.04	79.24 ± 45.54
No	20 (76.9)	0.09 ± 0.05	110.39 ± 99.95
Early progressor			
Yes	14 (53.8)	0.09 ± 0.05	121.15 ± 72.34
No	12 (46.2)	0.05 ± 0.01	55.12 ± 20.18

### Correlation of serum kyn/trp ratio and quinolinic acid concentration with immunotherapy response

**Fig. 1:**
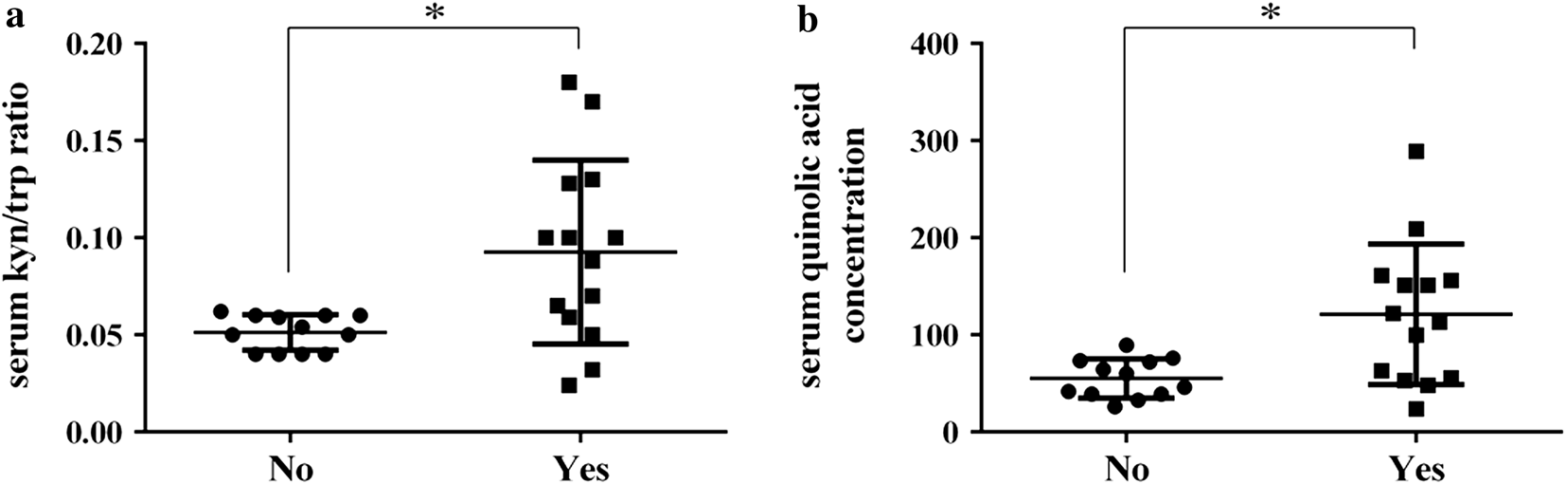
Correlation of serum kyn/trp ratio and serum quinolic acid concentration with immunotherapy response. Patients who showed an early progression (YES, n = 14) present a significantly higher concentration of kyn/trp ratio (**a**) and quinolic acid concentration (**b**) compared to patients who do not have an early progression (NO, n = 12). *#p *< 0.05 (Mann–Whitney test)

With a median follow up of 6.9 months, 7 (27%), 5 (19%) and 14 (54%) patients had a stable disease (SD), a partial response (PR) and a progressive disease (PD), respectively, according to i-RECIST v1.1 criteria. All patients were assessed at baseline for serum trp, kyn and quinolinic acid levels. Patients who showed an early progression (within 3 months) to nivolumab (n = 14) had a significantly higher concentration of kyn/trp ratio than others (0.09 vs 0.05, respectively, *p *= 0.01) as well as a higher quinolinic acid level (121,15 vs 55,12, respectively, *p *= 0.01) (Fig. 1).

On multivariate analysis (Table 2), kyn/trp ratio was significant associated with early progression (*p *= 0.01). We observed a significant correlation only between early progression and kyn/trp ratio and quinolinic acid concentration (respectively *p *= 0.017 and *p *= 0.005).

**Table 2:** Multivariate analysis: association between patiens characteristics and early progression

	*p value*	Odds ratio (95% CI)
Cerebral metastasis	0.48	3.18 (0.12–81.69)
Pleural effusion	0.66	0.53 (0.33–8.71)
ECOG PS	0.39	2.58 (0.92–22.75)
Serum kyn/trp	0.01	0.01 (0.00–0.41)
Serum quinolic acid	0.81	1.38 (0.09–20.30)

### Correlation of serum kyn/trp ratio and quinolinic acid concentration with survival

Median PFS was 4 months and median OS was 6.5 months in the whole cohort.

**Fig. 2:**
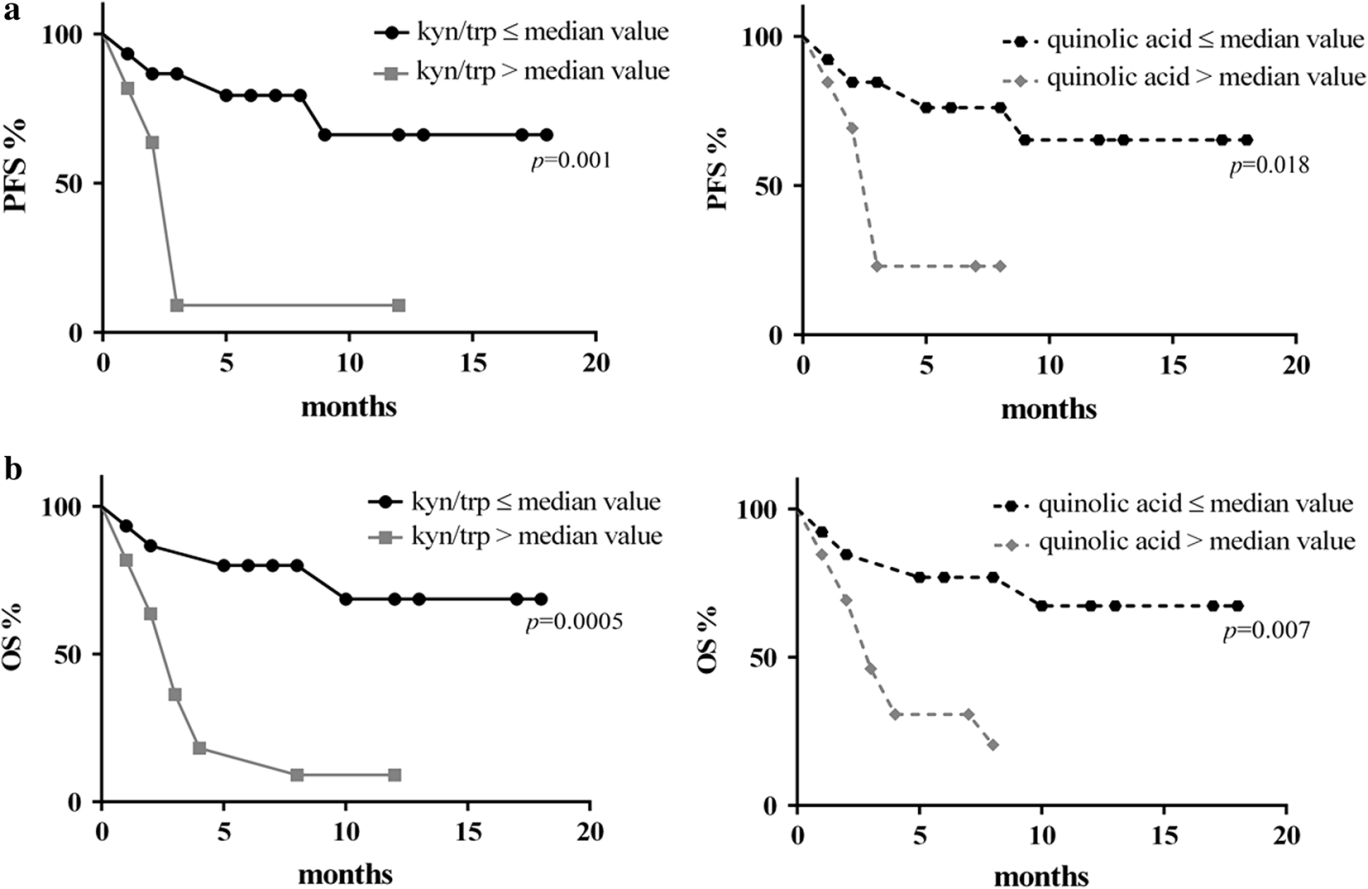
Correlation of serum kyn/trp ratio and quinolonic acid concentration with survival. Progression free survival (PFS, **a**) and overall survival (OS, **b**) were addressed by the Kaplan–Meier method and log-rank test

Patients were stratified by median value of serum kyn/trp value (0.06 µg/ml) and of serum quinolinic acid concentration (68.45 ng/ml). PFS was significantly longer in patients presenting lower values of kyn/trp than in patients showing higher values of kyn/trp (median PFS not reached versus 3 months; HR: 0.2; 95% CI 0.06–0.62; *p *= 0.001). Similar results were observed in patient stratified by quinolinic acid values (median PFS not reached versus median 3 months; HR: 0.3; CI 0.1–0.9; *p *= 0.018) (Fig. 2a).

Patients with a lower kyn/trp ratio value showed even a significantly better OS compared with patients with a higher kyn/trp ratio value (median OS not reached versus median 3 months; HR 0.18; 95% CI 0.06–0.56; *p *= 0.0005). Similar results were observed in patient stratified by quinolinic acid values (median OS not reached versus median 3 months; HR 0.2; 95% CI 0.06–0.660; *p *= 0.0077) (Fig. 2b).

## Discussion

In our preliminary analysis we found that baseline serum kyn/trp ratio, expression of IDO activity, could represent both a prognostic and predictive value in advanced NSCLC patients treated with second-line nivolumab. In fact, lower baseline level of kyn/trp ratio were significantly associated with better PFS and OS in our cohort. More interestingly and conversely, higher level of kyn/trp ratio correlated with early progression retaining an independent statistically significant value in multivariate analysis. In this study, patients were selected in accordance with eligibility criteria from registrative clinical trials of nivolumab in pretreated NSCLC (CheckMate 017 and CheckMate 057) and median PFS was consistent with historical data reported in the literature [16, 17].

Given the evidence that only a minority of cancer patients can benefit from ICI, a large amount of research is currently focused on defining predictive biomarkers able to select responsive or resistant patients in order to maximize the cost-effectiveness of treatment.

Over last few years, several predictive factors have been proposed that rely on patients’ characteristics as well as tumour and microenvironment profiles. PD-L1, which is expressed both on tumour and inflammatory cells, is arguably the most extensively studied biomarker for ICIs responsiveness, although it has showed some limitations [3]. More recently, the understanding of the correlation of microsatellite instability status with immunotherapy response has led to the FDA cancer site/histology-agnostic approval of the anti-PD-1 pembrolizumab [18]. Accordingly, tumour mutational burden (TMB) and mRNA profile are being considered promising response indicators [19]. In particular TMB has been used to select patients in CHECKMATE 227 trial, demonstrating a benefit of immunotherapy in patients with mutational load higher than 10 mutations per megabase. Regarding circulating biomarkers, some encouraging results have been reported for IL 8 [20] and circulating T lymphocytes [21]. However, all these putative biomarkers have strong limitations and they have not shown clinical usefulness for the different settings.

In this context, IDO has been identified as a marker of activation of immunological pathways induced by IFN-gamma, representing one of the main immune escape mechanism of tumor cells. IDO activity would also appear as negative prognostic factor in stage III-IV lung cancer [22–27].

Furthermore, Allison et al. demonstrated that IDO knockout mice treated with anti CTLA-4, anti PD-1 or anti GITR presented delay in B16 melanoma tumour growth and increased OS compared with wild-type mice [28]. Notably, tissue expression of IDO has been lately associated with a better response to nivolumab in patients with renal cell carcinoma [29].

Our preliminary results showed that kyn/trp ratio serum levels are statistically higher in early progressors allowing to identify a proportion of NSCLC patients with intrinsic resistance to anti PD-1, and with a poor prognosis both in terms of PFS and OS. In particular, the median PFS of the whole cohort is not reached, while the median PFS of “high kyn/trp ratio patients “was 3 months, very similar to that of hyperprogressive patients from the study by Champiat et al. [4]. Interestingly, in our cohort, the IDO activity does not seem to be correlated with clinical parameters such as brain metastases, pleural effusion, age and ECOG PS.

Moreover, quinolinic acid levels are strongly associated with response to nivolumab in our study. Quinolinic acid reflects the metabolism of kynurenine and it is involved in tumour progression not only diminishing immune activation but also increasing the synthesis of NAD, through quinolinic acid phosphoribosyltransferase, that is necessary to prevent apoptosis of neoplastic cells [30].

Our findings suggest a possible role to IDO in mediating resistance to anti PD-1 agents and provide a rationale for an early administration of anti-IDO compounds in those patients with high baseline kyn/trp ratio or high quinolinic acid levels. In fact, patient selection based on this biomarker could most probably avoid negative results of clinical trials designed without considering the immunological background of patients, i.e. the extent of the immunosuppressive milieu.

## Conclusion

In conclusion we found that the kyn/trp ratio and quinolinic acid level in the peripheral blood of NSCLC patients may be a potential predictive biomarker of resistance to anti-PD-1. The kyn/trp ratio and quinolinic acid are associated with early progression, PFS and OS. Despite the small sample, our study suggests that IDO could represent an easy-assessable and cost-effective biomarker particularly useful in identifying patients more likely to have a benefit with a combination of anti-PD-1 and anti-IDO. Future clinical trials are warranted to confirm its role as predictive biomarkers as well as therapeutic target, introducing immuno-oncology in the scenario of precision immuno-oncology.
